# A Novel Defined Endoplasmic Reticulum Stress-Related lncRNA Signature for Prognosis Prediction and Immune Therapy in Glioma

**DOI:** 10.3389/fonc.2022.930923

**Published:** 2022-06-30

**Authors:** Yinfei Zheng, Xiaoyu Yue, Cheng Fang, Zhuang Jia, Yuxiang Chen, Han Xie, Jiajia Zhao, Zhihao Yang, Lianxin Li, Zhigang Chen, Erbao Bian, Bing Zhao

**Affiliations:** ^1^ Department of Neurosurgery, The Second Affiliated Hospital of Anhui Medical University, Hefei, China; ^2^ Cerebral Vascular Disease Research Center, Anhui Medical University, Hefei, China

**Keywords:** glioma, lncRNA, endoplasmic reticulum stress, risk signature, prognosis, immune

## Abstract

Gliomas are a group of the most aggressive primary central nervous system tumors with limited treatment options. The abnormal expression of long non-coding RNA (lncRNA) is related to the prognosis of glioma. However, the role of endoplasmic reticulum (ER) stress-associated lncRNAs in glioma prognosis has not been reported. In this paper, we obtained ER stress-related lncRNAs by co-expression analysis, and then a risk signature composed of 6 ER stress-related lncRNAs was constructed using Cox regression analysis. Glioma samples in The Cancer Genome Atlas (TCGA) were separated into high- and low-risk groups based on the median risk score. Compared with the low-risk group, patients in the high-risk group had shorter survival times. Additionally, we verified the predictive ability of these candidate lncRNAs in the testing set. Three glioma patient subgroups (cluster 1/2/3) were identified by consensus clustering. We further analysed the abundance of immune-infiltrating cells and the expression levels of immune checkpoint molecules in both three subgroups and two risk groups, respectively. Immunotherapy and anticancer drug response prediction showed that ER stress-related lncRNA risk signature positively correlates with responding to immune checkpoints and chemosensitivity. Functional analysis showed that these gene sets are enriched in the malignant process of tumors. Finally, LINC00519 was chosen for functional experiments. The silence of LINC00519 restrained the migration and invasion of glioma cells. Hence, those results indicated that ER stress-related lncRNA risk signature could be a potential treatment target and a prognosis biomarker for glioma patients.

## Introduction

The central nervous system malignant tumors are one of the cancers with the worst prognosis, and glioma accounts for more than 3/4 of malignant brain tumors (1, 2). Gliomas are categorized into four grades (I~IV) grounded on histopathologic characteristics by the World Health Organization (WHO) (3, 4). In general, the higher the grade of glioma, the worse the prognosis. The median survival time of patients with grade IV glioma was only 13 months (5, 6). With the progression of medicine, the diagnosis and therapy of glioma have improved, but the prognosis of patients with glioma is still not ideal (7). In addition, the sensitivity and specificity of the biomarkers available for prognosis prediction of glioma patients are not high. Therefore, an accurate prognostic model and a new target for glioma treatment are needed.

LncRNA is an RNA transcript with more than 200 nucleotides that lacks the protein-coding ability (8). At present, more and more shreds of evidence show that lncRNAs play a considerable part in regulating transcription, RNA splicing, and mRNA translation (9). In recent years, the molecular biological functions of lncRNAs are being diffusely studied. Accumulating shreds of evidence suggest that aberrant expression of lncRNA plays importance in the genesis and the development of cancers. For instance, research has confirmed that XIST is notably increased in glioma tissues (10), whereas MEG3 can decrease the vitality of glioma cells and promote its apoptosis (11). At present, more and more studies showed that the dyregulated expression of lncRNA is a feature of gliomas and has a crucial role in the nosogenesis of gliomas. Additionally, the reduced expression of BCYRN1 increases the proliferation and migration of glioma cells, resulting in unfavorable outcomes in glioma patients (12).

ER stress is a stress response that restores the homeostasis of the ER, manifested as an unfolded protein response (UPR) (13). When cells can conquer ER stress, the UPR response is activated and induces pro-survival signaling, but when ER stress processing capacity is exceeded, UPR activates pro-death pathways (14). However, tumor cells, including glioma cells, usually can maintain UPR activation for a long time and support their survival. In addition, there is evidence that the ER stress response can drive the pro-inflammatory process of tumor cells, and the micro-environment formed by cancer cells and tumor immune cells helps tumor growth, progression, and metastasis (15–17). It is worth noting that the literature has reported that lncRNA and ER stress can regulate each other and jointly determine the fate of tumor cells (18). For example, studies have shown that lncRNA GOLGA2P10 prevents tumor cell apoptosis induced via ER stress by up-regulating BCL-Xl (19). Due to the heterogeneity of gliomas in genetic, epigenetic, and microenvironment, it would be much better to integrate multiple biomarkers into one model to better characterize gliomas and predict the prognosis of glioma patients (20).

In this research, glioma RNA sequencing data were obtained from the TCGA database and the Chinese Glioma Genome Atlas (CGGA) database, and then lncRNAs associated with ER stress were mined, which are strongly linked to the survival time of glioma patients. Ultimately, we identified a risk signature incorporating 6 ER stress-related lncRNAs in the TCGA cohort as an independent prognostic factor for glioma patients and validated it in the CGGA cohort. Subsequently, we further analyzed immune infiltrating cells and predicted immune checkpoints and chemotherapy sensitivity. At the same time, the pathways and biological processes regulated by ER stress-related lncRNAs were evaluated. In addition, consensus cluster analysis was utilized to identify three clusters of glioma patients to predict clinical outcomes. Finally, we silenced LINC00519 in glioma cells, which affected the biological functions of glioma cells. These results will help promote the development of targeted therapy for glioma.

## Methods

### Tissue Samples

Tissue samples were obtained from patients pathologically diagnosed with gliomas who had been treated by surgical resection in the Department of Neurosurgery of the Second Affiliated Hospital of Anhui Medical University (Hefei, China) with informed consent. All samples containing 6 normal samples and 17 glioma samples were frozen and stored at -80°C. The primary tumor characteristics and clinical information was shown in **Supplementary Table 1**. The present study was approved by the Research Ethics Committee of The Second Affiliated Hospital of Anhui Medical University.

### Data Source

TCGA RNA-seq data (689 samples) and clinical case samples (648 samples) in the training cohort were obtained from the TCGA database (http://cancergemome.nih.gov/). Meanwhile, CGGA RNA-seq data (618 samples) and clinical case samples (465 samples) in the testing cohort were obtained from the CGGA database (http://www.cgga.org.cn). 1132 normal brain tissue samples were downloaded from GTEx.

### Qualification of ER Stress-Related LncRNAs

We collected 108 ER stress-related genes from the literature. Pearson correlation analysis was performed utilizing the Limma R package, and the gene-lncRNA co-expression network was established under the standard of p<0.0001 and |correlation coefficient| >0.6. Then, a total of 161 ER stress-related lncRNAs were obtained, and the co-expression network was visualized by Cytoscape 3.8 software. Next, the R programming language was used to conduct differential analysis on normal and glioma tissues, and 24 differentially expressed lncRNAs were screened out.

### Establishment and Verification of ER Stress-Related Risk Model

First, univariate Cox regression analysis was conducted on 24 differentially expressed lncRNAs and sifted out 21 lncRNAs that were observably related to survival. Then, in order to further narrow the range of candidate lncRNAs, the least absolute shrinkage and selection operator regression analysis was performed using the glmnet R package. Finally, multivariate Cox regression analysis was utilized to establish a risk signature. The computational formula of risk scores was as follows:


$$Riskscore={\text{Σ}}_{\text{i}=1}^{\text{n}}coef(i)\times exp(i).$$

Exp(i) represented the relative expression level of each ER stress-related lncRNA, and Coef (i) represented the regression coefficient of each lncRNA computed by multivariate Cox regression analysis.

### Bioinformatics Analysis

In TCGA and CGGA databases, the median risk score was utilized to distinguish risk groups of glioma patients. Using survival R package to analyze the survival time of different risk groups and was visualized by Kaplan-Meier (K-M) survival curve. Cox regression analyses were utilized to determine whether the risk model is an independent prognostic factor for patients with glioma. Receiver operating characteristic (ROC) curves were utilized to appraise whether the signature is sensitive and specific to predict prognosis with the R package.

### Consensus Clustering

Based on the 6 selected ER stress-related lncRNAs, consensus clustering was carried out for patients in TCGA and CGGA databases using the Consensus Cluster Plus R package. The cumulative distribution function (CDF) and consensus matrix were utilized to estimate the best number of clusters.

### Estimation of the Immune Cell Abundance and Immune Checkpoint Molecules

Using the ESTIMATE algorithm to reckon the proportion of immune cells in the tumor microenvironment (TME) of each sample in the TCGA database, four scores including Stromal Score, Immune Score, Estimate Score, and Tumor Purity were used for quantification. The single sample gene set enrichment analysis (ssGSEA) algorithm was utilized to assess the abundance of 24 immune cells, and immune checkpoint molecules were evaluated using the ggpubr R package and visualized by boxplot using R language.

### Prediction of Response to Chemotherapy and Immunotherapy

Tumor immune dysfunction and exclusion (TIDE) algorithm (21) and subclass mapping (22) were utilized to assess clinical responses to programmed cell death ligand 1 (PD-1) and cytotoxic T lymphocyte-associated protein 4 (CTLA4) immune checkpoints in two risk groups in the TCGA database. The pRRophetic R package was utilized to calculate the semi-maximum inhibitory concentration (IC50) used to predict the treatment response of the TCGA cohort to five chemotherapeutic agents.

### Functional Analysis

The signaling pathways related to the six ER stress-related lncRNAs were shown by performing gene set enrichment analysis (GSEA). Using the clusterprofler R package to analyze the biological process of Gene Ontology (GO) enrichment and Kyoto Encyclopedia of Genes and Genomes (KEGG) pathway analyses.

### Cell Culture and Transfection

SNB19, SF126, LN18, T98G, U251, and SW1088 cell lines were purchased from the Chinese Academy of Sciences (Shanghai, China) and cultured in DMEM medium (Gibco, USA) containing 10% FBS (Gibco, USA). Glioma cells were cultured in an incubator containing 5% CO_2_ at 37°C. We used siRNA, designed by GenePharma Technology (Shanghai, China), to target and knockdown LINC00519. LINC00519 siRNA included three sequences (siLINC00519-1-forward GCCGCUGUGAUUUGGUAAATT, siLINC00519-1-forward UUUACCAAAUCACAGCGGCTT, siLINC00519-2-forward CUGGAGUGCAGUGACAUAATT, siLINC00519-2-forward UUAUGUCACUGCACUCCAGTT, siLINC00519-3-forward CACAAUAUGAGGGAAUUAUTT, siLINC00519-3-forward AUAAUUCCCUCAUAUUGUGTT).

When the cells were cultured to 60%-70% in a 6-well plate, replace the medium and 2mL DMEM medium containing 10% FBS was added for transfection. Next, transfection reagent JETPrime (Poly plus-transfection^®^) was utilized for cell transfection.

### RNA Extraction and RT-qPCR

Total RNA of tissues and cells was extracted using TRIzol reagent (Invitrogen, Thermo Fisher Scientific) in accordance with the protocol of the manufacturer. Then, PrimeScript RT Master Mix (RR036A; Takara) was utilized to perform reverse transcription. Subsequently, the TB Green™ Premix Ex Taq™ II (RR820A; Takara) was utilized to carry out RT-qPCR. Finally, the 2^−ΔΔCt^ method was performed to count the expression level of each lncRNA. The following PCR primers were used: GAPDH forward: 5’-CGCTCTCTGCTCCTCCTGT-3’; reverse: 5’- ATCCGTTGACTCCGACCTA -3’. LINC00519 forward: 5’-GCAGACACAACCACAGTTGG-3’; reverse: 5’- AGCTGCCCTTTGAAGCATTTC -3’.

### Migration and Invasion Experiment

Migration and invasion assays were performed using 24-well chambers (3422; Corning). For migration assay, 2×10^4^ transfected cells were re-suspended in serum-free medium and inoculated into the upper chamber, and 600µl medium containing 30% FBS was added to the lower chamber. And for the invasion assay, we added 1×10^5^ transfected cells to each chamber pre-coated with Matrix (356234; BD Biocoat). After being cultured for 24 (migration assay) or 48 (invasion assay) hours in 5% CO_2_ at 37°C, cells were fixed using 4% polyoxymethylene and stained using 0.5% crystal violet. Thereafter, cotton swabs were utilized to wipe cells remaining on the upper surface of chambers. Finally, we counted and photographed the cells under an inverted microscope.

### Analysis of Data

RStudio and GraphPad Prism 9.0.0 software were utilized to analyze and visualize data. Transwell cells and clone colonies were counted by ImageJ software. Student’s t-test and Chi-square test were utilized for statistical analysis. P-value threshold is two-tailed and p<0.05 was considered statistically significant. All cell experiments were repeated three times. *p<0.05, **p <0.01, ***p < 0.001.

## Results

### Differential Expression Profiles and Screening of ER Stress-Related LncRNAs

**Figure 1:**
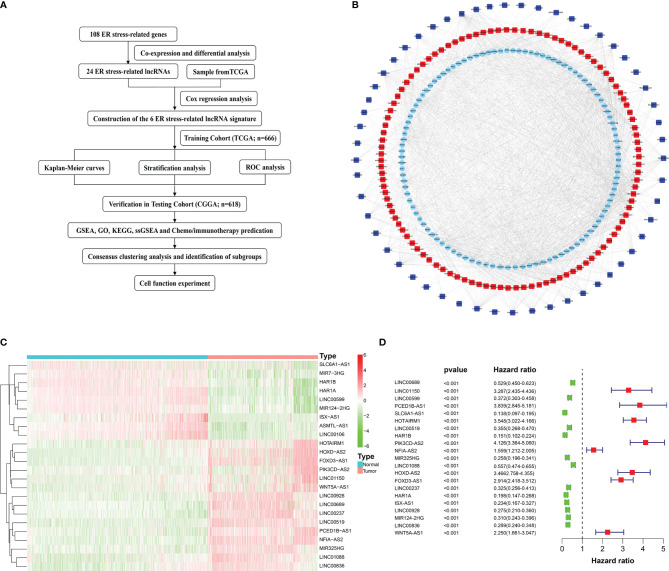
Flow diagram and the acquisition, identification, and screening of ER stress-related lncRNAs. **(A)** Flow chart of this study. **(B)** ER stress-related lncRNAs and ER stress-related genes interaction network. Squares represent ER stress-related lncRNAs and circles represent ER stress-related genes. Red indicates positive correlation and blue indicates negative correlation. **(C)** Heatmap of 24 differentially expressed ER stress-related lncRNAs. **(D)** Univariate Cox regression analysis.

The flow chart of our study was shown in (**Figure 1A**). 161 ER stress-related lncRNAs were obtained by establishing a co-expression network with 108 ER stress-related genes (**Figure 1B**). Subsequently, 24 differentially expressed lncRNAs were identified in normal and glioma tissues (**Figure 1C**). Eventually, 21 ER stress-related lncRNAs were filtrated through univariate Cox regression analysis (**Figure 1D**).

### Identification of ER Stress-Related LncRNA and Construction of Risk Signatures

**Figure 2:**
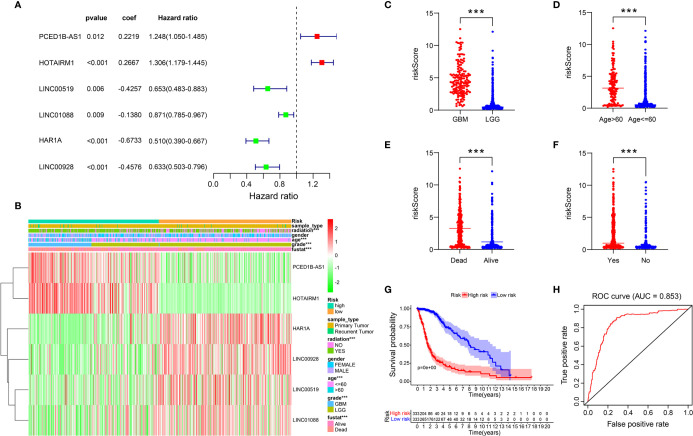
A risk model with the 6 selected ER stress-related lncRNAs. **(A)** Multivariate Cox regression calculated the hazard ratio (HR) and regression coefficient of the six ER stress-related lncRNAs. **(B-F)** Differences in risk scores and clinicopathological features between two risk groups. **(G)** The K-M curves of two risk groups in training cohort. **(H)** ROC curve indicates the sensitivity and specificity of predicting the OS of patients in the training cohort.

To construct a risk model, multivariate Cox regression analysis was conducted on 21 ER stress-related lncRNAs, out of which 6 lncRNAs (PCED1B-AS1, HOTAIRM1, HAR1A, LINC00928, LINC00519, LINC01088) associated with prognosis were selected (**Figure 2A**). Based on the median risk score, the training cohort was split into two risk groups (**Figure 2B**). Simultaneously, we found that the high-risk group and the low-risk group have significant differences in WHO grade (**Figure 2C**), patient age (**Figure 2D**), survival status (**Figure 2E**), and radiation (**Figure 2F**). In addition, we also perceived that the OS of glioma patients in the high-risk group was lower (**Figure 2G**). The ability of the risk signature to predict prognosis was then assessed using the ROC curve. The area under the curve of our risk model was 0.853 (**Figure 2H**).

### Verification of the Prognostic Signature

**Figure 3:**
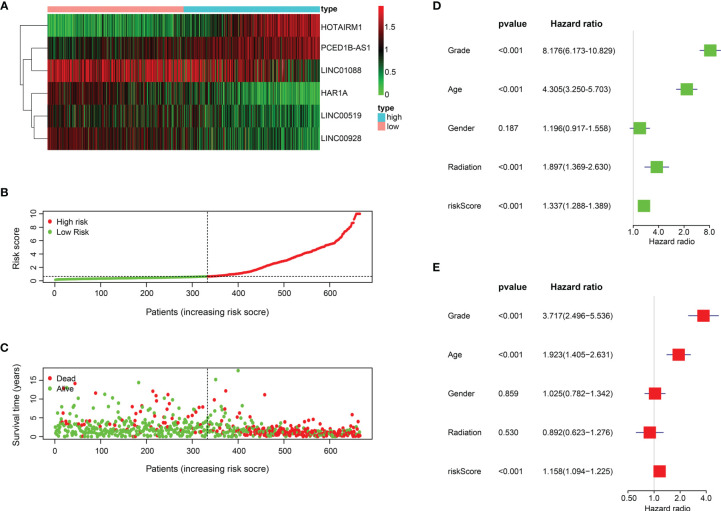
The prognostic value of the 6 ER stress-related lncRNAs. **(A)** 6 ER stress-related lncRNA expression profiles. **(B, C)** Distribution of survival status, OS, and risk score in training set. **(D, E)** Univariate **(D)** and multivariate **(E)** Cox regression analyses in training set.

Based on the six ER stress-related lncRNAs signature, patients in the training set were separated into two groups. The expression levels of 6 lncRNAs in two risk groups are shown in the heatmap in **Figure 3A** (**Figure 3A**). **Figures 3B, C** show the risk scores and survival status distribution of the two risk groups (**Figures 3B, C**). Univariate Cox regression analysis manifested that the risk score, age, radiation, and WHO classification of glioma patients were related to OS (**Figure 3D**). Multivariate Cox regression analysis exhibited that risk score, age, and WHO classification are correlated to OS (**Figure 3E**). The results of the training cohort indicate that the newly constructed risk model is a better predictor of prognosis.

### Validation of Prognostic Signature in Testing Cohort

**Figure 4:**
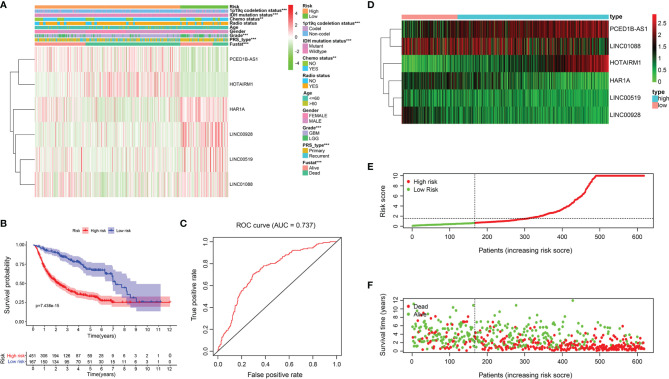
Relationship between risk model and clinicopathological features and prognostic value in CGGA cohort. **(A)** Heatmap showed the expression level of 6 ER stress-related lncRNAs and the distribution of clinicopathological features in risk subgroups. **(B)** K-M survival analysis based on the 6 ER stress-related lncRNAs between two risk groups in testing cohort. **(C)** ROC curve evaluates the predictive efficiency of the risk model in the testing database. **(D)** Expression profiles of six ER stress-related lncRNAs in risk subsets in glioma patients. **(E, F)** Distribution of risk score, survival time, and survival status of glioma patients in testing set.

Based on the risk model built on the training set, the risk of the test cohort (CGGA database) is scored. Similar to the training set, glioma samples in the testing cohort were separated into two risk subgroups (**Figure 4A**). K-M curve revealed that patients in the high-risk group had lower OS (**Figure 4B**). The ability to predict of OS was assessed by ROC, and the area under the curve was 0.737 (**Figure 4C**). **Figures 4D-F** respectively show lncRNA expression profile, risk score distribution, and survival status distribution between the two risk groups (**Figures 4D-F**).

### Clinicopathological Features and Immune Microenvironment of Three Clusters of Patients With Glioma

**Figure 5:**
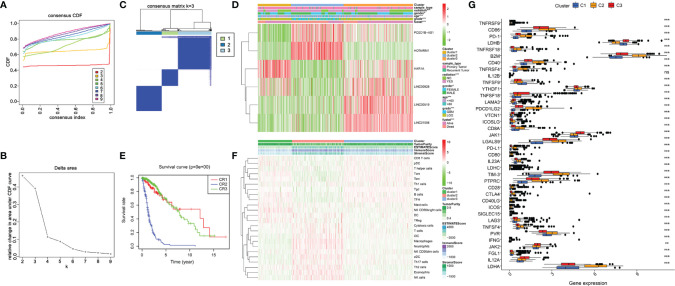
Clinicopathological characteristics and immune microenvironment in consensus clustering subgroups of gliomas. **(A)** Consensus clustering CDF for k = 2 to 9. **(B)** Of the length and slope of the CDF curve as the index changes from 2 to 9. **(C)** The consensus score matrix of all samples when k= 3. (**D**)Heatmap of ER stress-related lncRNAs between three clusters of the TCGA cohort. **(E)** K-M curves showed differences in OS among three subclusters in the training set. **(F)** The enrichment levels of 24 immune-related cells in three subgroups. **(G)** The boxplot shows the expression of 38 immune checkpoint molecules between the three subgroups. **p < 0.01, ***p < 0.001.

To determine the molecular subtype, we performed a consistent clustering of 6 prognostic lncRNAs related to ER stress. Considering CDF and clinical significance, we choose k=3 as the appropriate number of clusters for further analysis. Finally, glioma samples were divided into cluster 1, cluster 2, and cluster 3 (**Figures 5A-C**). Next, we compared the clinical and molecular characteristics of the three subgroups. Cluster 2 is significantly related to WHO grade, age, radiation, living conditions, and gender (**Figure 5D**). In addition, we also found that patients in cluster 2 had the shortest OS (**Figure 5E**). These results have also been verified in the CGGA database (**Figure S1**).

ER stress is closely associated with immunity, thus, we downloaded the expression profile of 666 glioma patients to evaluate their immune status and tried to assess the function of immune cells in the malignant progression of glioma (1). Subsequently, we analyzed the infiltration of immune cells in three subgroups by the ssGSEA algorithm. The result exhibited that Macrophages, Neutrophils, NK cells, T cells, Th17 cells, and Th2 cells were enriched in cluster 2 (**Figure 5F**). Additionally, we assessed the expression of immune checkpoint molecules in three clusters. Results exhibited high expression of PD-1, CTLA4, TIM-3, PD-L1, LAG3, CD86, CD80, and CD28 in cluster 2 (**Figure 5G**). In conclusion, the cluster subgroups showed distinct clinicopathological features and immune microenvironment, and the changes in the immune microenvironment may be related to the shorter OS time of cluster 2.

### Tumor Immune Microenvironment of Glioma in Two Risk Groups

**Figure 6:**
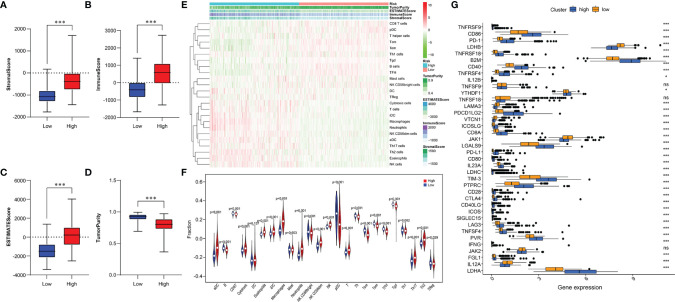
Relationship between immune infiltration and immune checkpoint and risk model. **(A-D)** Comparison of Stromal Score **(A)**, Immune Score **(B)**, ESTIMATE Score (**C**), and Tumor Purity **(D)** between two risk subgroups. **(E)** The enrichment levels of 24 immune cells in two risk groups. **(F)** Violin plot displayed the different proportions of tumor-infiltrating cells between the two risk groups (blue was the low-risk group, and red was the high-risk group). **(G)** The boxplot shows the expression of 38 immune checkpoint molecules between the two risk subgroups. *p < 0.05, ***p < 0.001; NS, no significance.

Given the distinct immunological infiltrations shown in three subgroups, we chose to further assess immunological status in two risk groups. Thus, indicators evaluating the TME of each sample were scored using the ESTIMATE algorithm, and the TME characteristics in two risk groups were compared. The results indicated that the high-risk group had a higher Stromal Score, Immune Score, and ESTIMATE Score, and lower Tumor Purity (**Figures 6A-D**). Using the ssGSEA algorithm, we analyzed the abundance of immune cells in two risk groups. The result has shown that Macrophages, T cells, Th17 cells, Th2 cells, Cytotoxic cells, Neutrophils, and NK cells were enriched in the high-risk group. Contrarily, B cells, CD8^+^T cells, Th cells, Th1 cells, and Treg cells were enriched in the low-risk group (**Figures 6E, F**). Additionally, we assessed the correlation between risk characteristics and immune checkpoint molecules. Results showed high expression of PD-1, CTLA4, TIM-3, PD-L1, LAG3, CD86, CD80, and CD28 in the high-risk group, and high expression of LDHB, LAMA3, VTCN1, JAK1, and IL12A in the low-risk group (**Figure 6G**). We found that risk signature can be utilized to predict the cellular immune characteristics of gliomas.

### Prediction for Immunotherapy and Anticancer Drug Response

**Figure 7:**
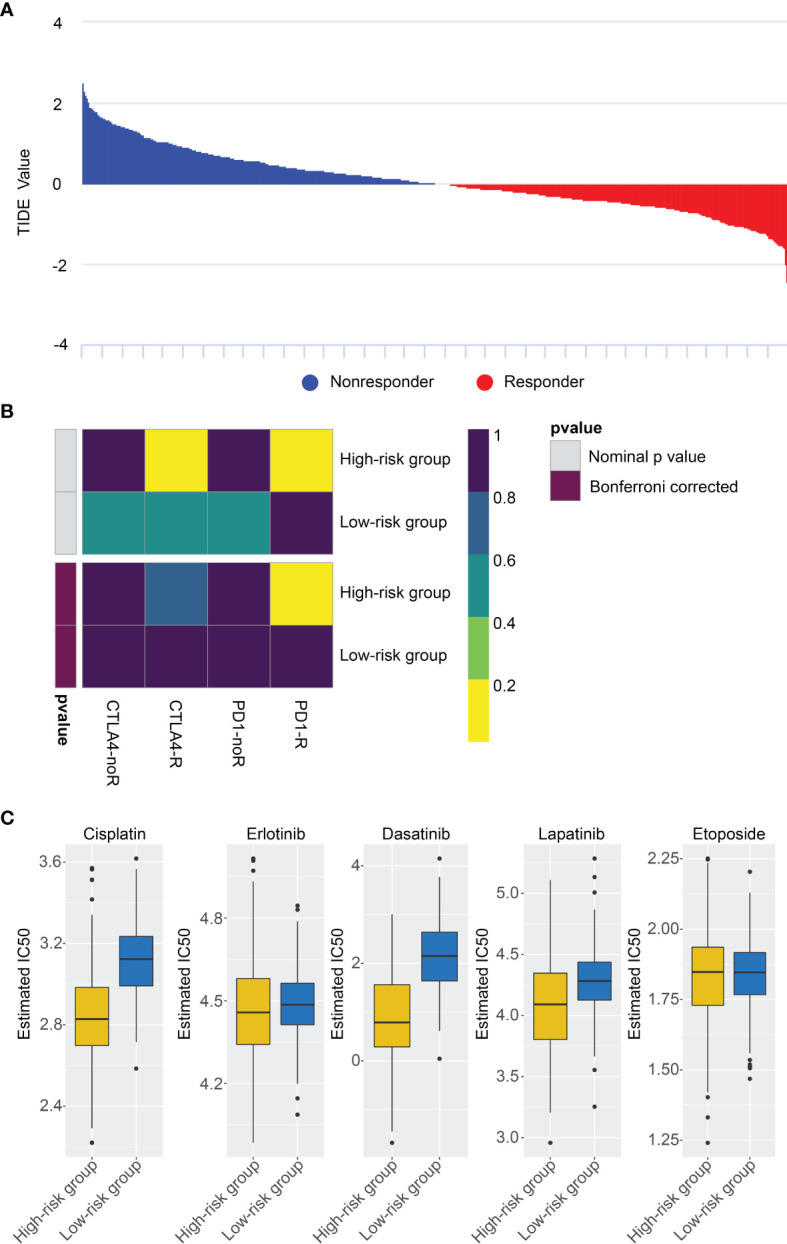
Differential putative chemo/immunotherapeutic response. **(A)** TIDE value and response results to immunotherapy of patients with glioma. **(B)** Submap analysis showed that high-risk group may be more sensitive to anti-CTLA-4 and anti-PD-1 therapy. **(C)** Estimated IC50 indicates the efficiency of chemotherapy to the two risk groups by Cisplatin, Erlotinib, Dasatinib, Lapatinib, Etoposide.

We investigated the capacity of the risk signature to distinguish between patients who respond differently to immune checkpoint blocking therapy (**Figure 7A**). We found that anti-PD1 and anti-CTLA4 immunotherapy had better clinical efficacy in the high-risk group (**Figure 7B**). Currently, chemotherapeutic drugs are commonly used to treat gliomas, so we attempted to assess the response of these two risk subtypes to commonly used drugs. Therefore, ridge regression was utilized to train the prediction model on the GDSC cell line data set and evaluate the prediction accuracy of ten-fold cross-validation. According to the prediction model of 5 chemotherapeutics, the IC50 value of each sample is calculated in the TCGA data set. We found high-risk group may be more sensitive to frequently used chemotherapeutic drugs (Cisplatin, Erlotinib, Dasatinib, Lapatinib, Etoposide) (**Figure 7C**).

### Functional Analysis of the Risk Signature

**Figure 8:**
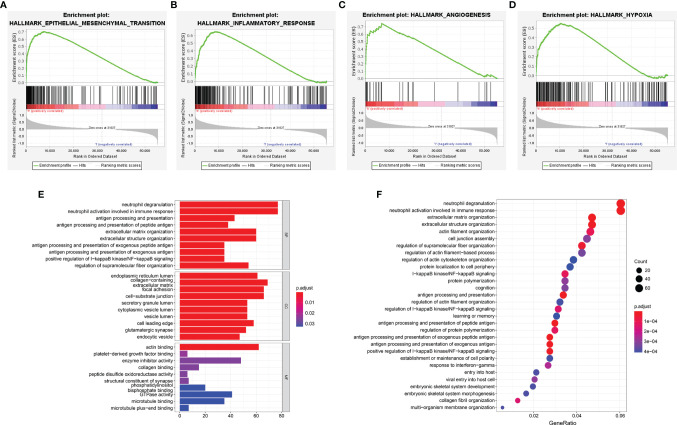
Functional enrichment analyses of the risk signature. **(A–D)** GSEA analysis in TCGA cohort. **(E, F)** The GO enrichment **(E)** and KEGG **(F)** pathway analysis.

To probe the underlying changes of functional characteristics associated with the 6 ER stress-related lncRNAs, GSEA, GO, and KEGG analyses were performed between the two risk groups. GSEA analysis showed that the high-risk group was closely bound up with mesenchymal transition, inflammatory response, angiogenesis, and hypoxia (**Figures 8A-D**). GO enrichment analysis showed that the positively related genes were mainly enriched in neutrophil activation and antigen processing and presentation related to immune response, positive regulation of I-κB kinase/NF-κB signaling, and cell-matrix adhesion related to malignant progression of glioma (**Figure 8E**). Next, the terms of KEGG pathway were I-κB kinase/NF-κB signaling, extracellular structure organization, and antigen processing and presentation (**Figure 8F**). These results suggest that the risk signature is related to the malignant biological processes of gliomas.

### Silencing of LINC00519 Restrained the Migration and Invasion of Glioma Cells

**Figure 9:**
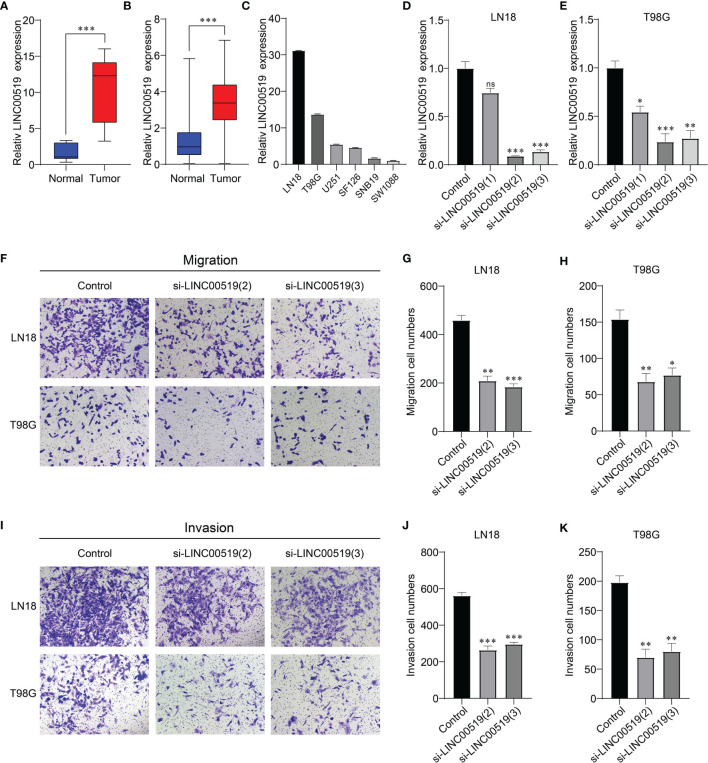
Verification of the silencing effect. **(A)** Relative expression levels of LINC00519 in normal and glioma brain tissues from our cohort. **(B)** Relative expression levels of LINC00519 in normal and glioma tissues from GTEx (n=1132) and the TCGA database (n=689). **(C)** Relative expression of LINC00519 in six cell lines. **(D, E)** Detection of the relative silencing levels of LINC00519 in LN18 **(D)** and T98G **(E)** cells. **(F–H)** Representative imaging **(F)** or counting **(G, H)** of migration assays after silencing with LINC00519 in LN18 and T98G cells. **(I-K)** Representative imaging **(I)** or counting **(J, K)** of invasion assays after silencing with LINC00519 in LN18 and T98G cells. *p < 0.05, **p < 0.01, ***p < 0.001; NS, no significance.

To further investigate the role of those 6 ER stress-related lncRNAs in glioma, we analyzed these six lncRNAs and found that LINC00519 is less studied in gliomas and has not been tested for function. Therefore, we selected LINC00519 for functional analysis in glioma. Firstly, we observed LINC00519 expression in glioma tissue versus normal brain tissue from our cohort. **Figure 9A** showed that the expression of INC00519 is significantly increased in glioma tissue compared with normal brain tissue (**Figure 9A**). Likewise, high expression of LINC00519 in glioma tissue versus normal brain tissues was confirmed from integrated analyses of the TCGA and GTEx databases (**Figure 9B**). Then, we detected the expression levels of LIC00519 in glioma cell lines by RT-qPCR. The expression levels of LINC00519 in LN18 and T98G are higher than in other cell lines (**Figure 9C**). We then performed knockdown of LINC00519 in LN18 and T98G cells with siRNA and detected the knockdown efficiency with RT-qPCR. The results showed that si-LINC00519 sequences 2 and 3 had better knockout efficiency in both LN18 and T98G cell lines (**Figures 9D, E**). Finally, the cell transwell experiment manifested that silencing of LINC00519 impaired the migration and invasion ability of glioma cells (**Figures 9F–K**). These results suggest that LINC00519 may be a new target for glioma.

## Discussion

Glioma is the most pernicious primary tumor in the central nervous system due to its rapid malignant progression and high recurrence rate (23, 24). Because of the molecular heterogeneity of glioma, the prognosis is extremely poor. The traditional WHO grading does not accurately distinguish between subgroups of cancer risk associated with different clinical outcomes (25). An in-depth understanding of glioma genome changes has facilitated the discovery of prognostic characteristics to facilitate individualized treatment decisions (26–28). At present, although there is a deeper cognition of the molecular mechanism of gliomagenesis and development, there is still a lack of molecular markers that can accurately predict the prognosis and guide the therapy of glioma (29). Hence, the underlying prognostic and therapeutic targets for glioma need to be urgently explored.

Sequencing of the human genome has resulted in the discovery of a large number of transcripts of genes called lncRNA that do not have protein-coding capabilities. Studies have shown that in many human malignancies, including glioma, functional alterations of specific lncRNAs promote tumor formation, progression, and metastasis (30). Previously, our study found that the activation of lncRNA TMEM44-AS1 can promote malignant progression of glioma through Myc positive feedback loop (31). Tumor cells often grow in an anoxic environment with chronic ER stress, which is caused by the accumulation of misfolded and unfolded proteins in the ER under various stimuli and pathological conditions (32, 33). It is not clear how cancer cells escape ER stress and survive under harsh conditions, and the pathogenic role of lncRNA in the regulation of ER stress remains to be explored (19). The prognosis and treatment of tumors can be predicted based on cancer-related molecular markers, which were widely researched in recent years (34). A risk model based on 8 lncRNA serves as an independent prognostic biomarker of esophageal squamous cell carcinoma and may come into play in evaluating the response of the immune checkpoints to block immunotherapy for esophageal squamous cell carcinoma (35). We previously constructed a signature comprising 9 lncRNA methylated genes that may afford a new predictive and therapeutic target for glioma patients (9). At present, the role of lncRNA related to ER stress in the prognosis of glioma has not been studied. So, here we built a risk model with 6 lncRNAs to predict the prognosis of glioma patients.

In this research, multiple ER stress-related lncRNAs were integrated into one risk signature to evaluate whether the risk signature is a stronger predictor of glioma prognosis. Firstly, an alternative prognostic model consisting of 6 ER stress-related lncRNAs was constructed. Subsequently, we used two databases, TCGA and CGGA, to test and verify the accuracy and predictive performance of the risk signature in assessing the prognosis of glioma patients. The results showed that this risk signature had a good effect on predicting the prognosis of patients with glioma. In addition, based on the expression level of the 6 ER stress-related lncRNAs, we identified three glioma subgroups, cluster 1, cluster 2, and cluster 3. The cluster 1/2/3 subgroups exhibited different prognoses and clinicopathological features. Therefore, in future work, the risk score of glioma patients can be calculated in accordance with the risk model we constructed, and speculate the prognosis based on the calculated risk score.

Functional analyses revealed that biological processes of immune and inflammatory responses were mainly enriched in high-risk populations, suggesting an interaction between the ER stress-related lncRNA signature and the glioma immune response. Previous studies have manifested that ER stress can play a significant role in tumor development through its immunomodulatory function (36). The proportion of tumor cells in TME reflects tumor purity, while glioma cases with lower purity have a more obvious malignant tendency, which often leads to poor prognosis (37). In our study, the high-risk group showed lower tumor purity, while stromal and immune scores were markedly elevated. Tumor-infiltrating immune cells play multiple roles in the progression of glioma and the immune checkpoint molecules are often up-regulated in the TME of various malignancies (38, 39). In this paper, we compared the abundance of immune cells and the expression of multiple immune checkpoint molecules in three subgroups and two risk groups. PD-1 is normally upregulated on activated T cells and NK cells to induce immune tolerance (40, 41). Enrichment of CD8^+^T cells, representing antitumor cells, obviously extends patient survival (42). And tumor cells often overexpress programmed cell death ligand 1 (PD-L1) which contributes to immune escape (43). PD-1 has also previously been shown to be expressed on tumor-associated macrophages (TAMs), limiting their phagocytosis (44). TAMs can also promote tumor progression to malignancy by promoting tumor cell invasion, migration, intravascular infiltration, and inhibiting anti-tumor immune responses (45–47). CTLA4 is an immune checkpoint co-inhibitory receptor mainly expressed on T cells (48). CTLA4 can compete with the co-stimulatory receptor CD28 for binding to CD80 and CD86 ligands, and can inhibit the activation of Th cells (49). The upregulation of PD-1 on Th cells can inhibit immune response and the expression of lymphocyte-activation gene-3 (LAG3), an inhibitory molecule, can promote the suppression of Th cells (50). The imbalance of Th1/Th2 cell ratio is also the key to the malignant progression of tumors. Generally speaking, Th1 cells kill tumor cells by secreting interferon γ, while Th2 cells produce cytokines that promote tumor development (51). Past research has shown that T cell immunoglobulin-3(TIM-3) restrains Th1 cell reactions employing the TIM-3/galectin-9 pathway, generating an inhibitory TME and immune escape (52, 53). Consistent with these conclusions, in our study, the cluster 2 group and the high-risk group had a higher abundance of macrophages, NK cells, T cells, Th2 cells, and Th17 cells and a higher expression of PD-1, PD-L1, CTLA4, CD86, CD80, CD28, LAG3, and TIM-3, while the abundance of Th cells and Th1 cells was lower.

TIDE is considered to be highly effective in predicting the efficacy of immune checkpoint inhibitors. The use of immunosuppressants of PD-1 and CTLA-4 to treat malignant tumors has also been successful in a variety of tumors, but the efficacy in gliomas has been unsatisfactory. Therefore, the TIDE score was selected to assess the efficacy of PD1 and CTLA4 immunotherapy in the two risk groups. Our results show that the high-risk patients may respond well to immune checkpoint therapy (PD-1, CTLA4) and benefit from immunotherapy. Subsequently, we revealed the sensitivity of high-risk and low-risk patients to five chemotherapeutic drugs (Etoposide, Cisplatin, Erlotinib, Dasatinib, Lapatinib) based on the consequences of database analysis, and determined that high-risk group patients had a better response to chemotherapy, which would provide a new way for researchers to develop more effective chemotherapy drugs. The above discussion indicates that the heterogeneity of tumor immune-microenvironment would generate different responses to immunotherapy or anti-cancer drugs, and our study may provide a direction for the treatment of glioma.

Solid tumors, such as glioma, often grow in a micro-environment characterized by hypoxic (54). This condition contributes to the increase in the amount and activity of HIF-1α and the occurrence of ER stress (55). Studies have shown that HIF-1α can promote the malignant progression of tumors through various mechanisms, such as tumor cell invasion, angiogenesis, and immune suppression (56). The occurrence of ER stress contributes to the dissociation of the ER chaperone glucose regulatory protein 78 (GRP78), thereby blocking its downstream effectors, and eventually leading to the occurrence of epithelial-mesenchymal transition (EMT) (57–59). EMT is one of the most common ways for epithelial tumors to increase their malignant behavior and disease progression (60). NF-κB, a transcriptional regulator, directly or indirectly triggers the release of proinflammatory mediators and leads to the infiltration of neutrophils and macrophages (61). Our GSEA results show that the high-risk group was enriched in inflammation-related pathways and malignant biological processes such as hypoxia, angiogenesis, and EMT, which are consistent with these findings. Similarly, GO and KEGG analysis indicated that ER stress-related lncRNAs were closely correlated with inflammation-related pathways, tumor cell adhesion, and NF-κB signaling. Since the high-risk group is related to the malignant progression of cancers, we selected LINC00519 for migration and invasion experiments in glioma cell lines. Transwell assay confirmed that silencing of LINC00519 restrained the migration and invasion of glioma cells. However, in-depth research on the exact molecular mechanisms of these 6 ER stress-related lncRNAs in the effect on gliomas was needed, and we need to further study the role of ER stress-related lncRNA in gliomas in animal models.

To sum up, based on the 6 screened ER stress-related lncRNAs, we constructed a risk signature, which can predict the prognosis of glioma patients, and we identified 3 subgroups of glioma patients with different prognostic and clinical characteristics. Meanwhile, the different immune characteristics of glioma patients in the two risk groups were exhibited. Functional analysis showed that ER stress-related lncRNAs could affect the migration and invasion of glioma cells. In conclusion, our study may have directive value for clinicians to analyze the prognosis of patients with glioma and realize individualized treatment.

## Data Availability Statement

The original contributions presented in the study are included in the article/**Supplementary Material**. Further inquiries can be directed to the corresponding authors.

## Ethics Statement

The studies involving human participants were reviewed and approved by the Ethics committee of the Second Affiliated Hospital of Anhui Medical University. The patients/participants provided their written informed consent to participate in this study.

## Author Contributions

BZ and EB designed this article. YZ and XY implemented the experiments. CF, ZJ, and YC collected the data. HX, JZ, and ZY screened and checked the data. LL and ZC drafted the manuscript. BZ and EB revised the manuscript and were responsible for the whole study. All authors made substantial contributions to the study and provided the approval of the submitted version.

## Funding

This research was funded by the National Natural Science Foundation of China (No. 81972348), Key Research and Development Plan Project of Anhui Province (No. 31804h08020270), College Excellent Youth Talent Support Program in Anhui Province (No. gxypZD2019019), Key Projects of Natural Science Research in Anhui Province (KJ2019A0267), Academic Funding Project for Top Talents in Colleges and Universities in Anhui Province (No. gxbjZD10), Nova Pew Plan of the Second Affiliated Hospital of Anhui Medical University (No. 2017KA01), Open Projects of Key Laboratory in Medical Physics and Technology of Anhui Province (LHJJ202001).

## Conflict of Interest

The authors declare that the research was conducted in the absence of any commercial or financial relationships that could be construed as a potential conflict of interest.

## Publisher’s Note

All claims expressed in this article are solely those of the authors and do not necessarily represent those of their affiliated organizations, or those of the publisher, the editors and the reviewers. Any product that may be evaluated in this article, or claim that may be made by its manufacturer, is not guaranteed or endorsed by the publisher.

## Supplementary Material

Supplementary Figure 1
**|** Clinical and molecular characteristics in consensus clustering subgroups of gliomas in the testing group. **(A)** Consensus clustering CDF for k = 2 to 9. **(B)** Of the length and slope of the CDF curve as the index changes from 2 to 9. **(C)** Consensus matrix for the two groups from the CGGA database **(D)** Heatmap of ER stress-related lncRNAs between three clusters of the CGGA cohort. **(E)** K–M survival curve.

## Abbreviations

lncRNA, long non-coding RNA; ER, endoplasmic reticulum; TCGA, The Cancer Genome Atlas; WHO, the World Health Organization; UPR, unfolded protein response; CGGA, the Chinese Glioma Genome Atlas; ROC, receiver operating characteristic; CDF, cumulative distribution function; GSEA, Gene set enrichment analysis; GO, Gene Ontology; KEGG, Kyoto Encyclopedia of Genes and Genomes; TME, tumor microenvironment; ssGSEA, the single sample gene set enrichment analysis; TIDE, Tumor immune dysfunction and exclusion; IC50, inhibitory concentration; OS, overall survival; K-M, Kaplan Meier; GDSC, Genomics of Drug Sensitivity in Cancer; PD-1, programmed cell death protein 1; PD-L1, programmed cell death ligand 1; TAM, tumor-associated macrophage; NK, nature killer; CTLA4, cytotoxic T lymphocyte-associated protein 4; LAG3, lymphocyte-activation gene-3; TIM-3, T cell immunoglobulin-3; HIF-1α, hypoxia-inducible factor-1α; GRP78, glucose regulatory protein 78; EMT, epithelial-mesenchymal transition; HR, hazard ratio; XIST, X-inactive specific transcript; MEG3, maternally expressed 3; BCYRN1, brain cytoplasmic RNA 1; FBS, fetal bovine serum.
