# Supplementary figures and images for: A Novel Defined Endoplasmic Reticulum Stress-Related lncRNA Signature for Prognosis Prediction and Immune Therapy in Glioma
Source: Front Oncol. 2022 Jun 30;12:930923. doi: 10.3389/fonc.2022.930923 (PMC9282894; doi:10.3389/fonc.2022.930923)

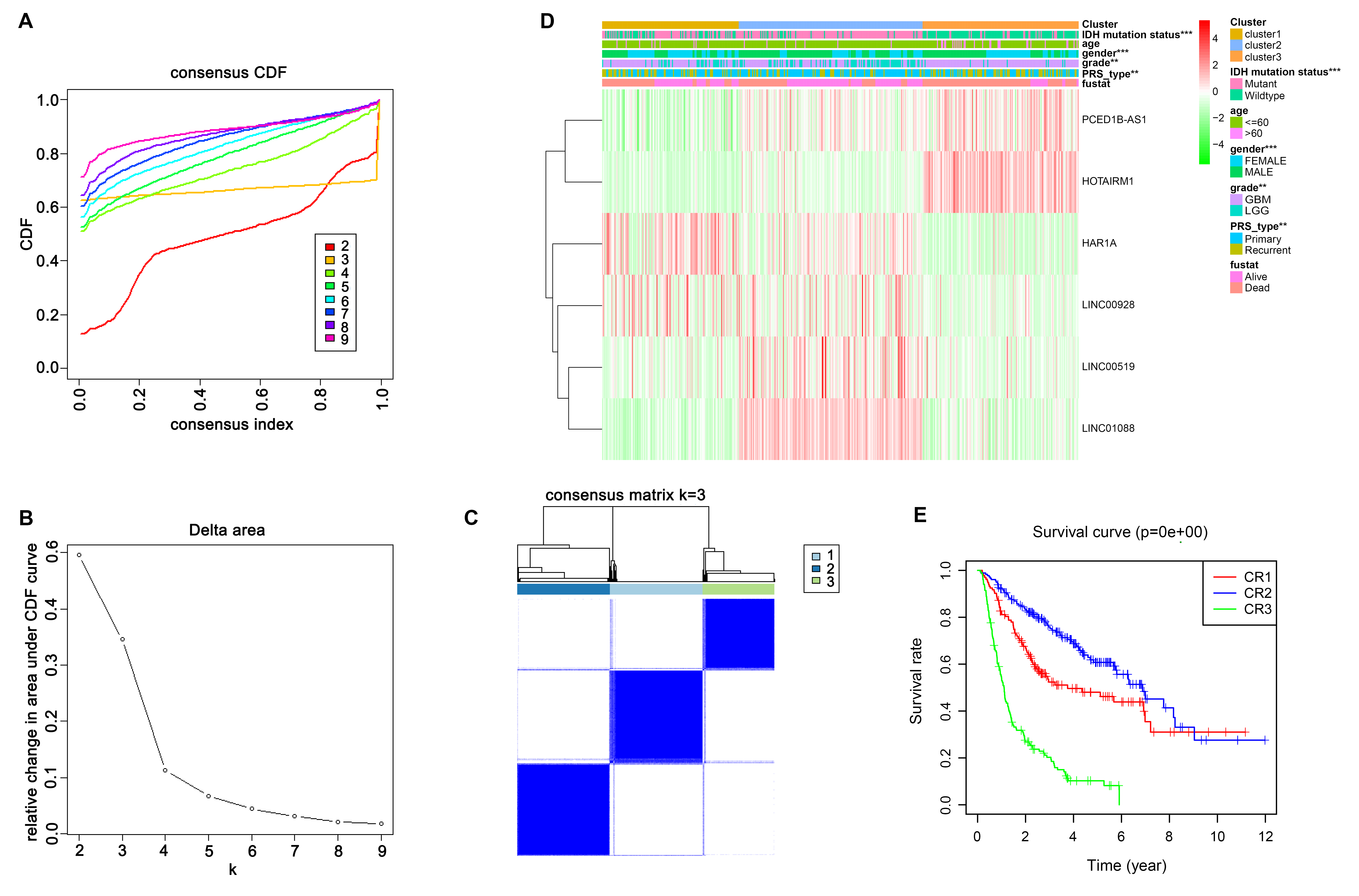

Supplement: Supplementary Figure 1 — | Clinical and molecular characteristics in consensus clustering subgroups of gliomas in the testing group. (A) Consensus clustering CDF for k = 2 to 9. (B) Of the length and slope of the CDF curve as the index changes from 2 to 9. (C) Consensus matrix for the two groups from the CGGA database (D) Heatmap of ER stress-related lncRNAs between three clusters of the CGGA cohort. (E) K–M survival curve. [file Image_1.jpeg]
