# Supplementary material for: A Novel Defined Endoplasmic Reticulum Stress-Related lncRNA Signature for Prognosis Prediction and Immune Therapy in Glioma
Source: Front Oncol. 2022 Jun 30;12:930923. doi: 10.3389/fonc.2022.930923 (PMC9282894; doi:10.3389/fonc.2022.930923)
Supplement: Supplementary file 2 [file Table_1.docx]

**Supplementary Table 1** Clinic-pathological characteristics of 17 glioma patients

| Characteristic | number of patients |
| --- | --- |
|  |  |
| KPS |  |
| >80 | 11 |
| <80 | 6 |
| Gender |  |
| Male | 8 |
| Female | 9 |
| Age |  |
| ≤ 50 | 5 |
| > 50 | 12 |
| Grade |  |
| Low grade | 7 |
| GBM | 10 |
